# Characterisation of a cell-free synthesised G-protein coupled receptor

**DOI:** 10.1038/s41598-017-01227-z

**Published:** 2017-04-24

**Authors:** Patrick J. Shilling, Fabian Bumbak, Daniel J. Scott, Ross A. D. Bathgate, Paul R. Gooley

**Affiliations:** 1 Department of Biochemistry and Molecular Biology, The University of Melbourne, Parkville, VIC 3010 Australia; 2 Bio21 Molecular Science and Biotechnology Institute, 30 Flemington Road, The University of Melbourne, Parkville, VIC 3010 Australia; 3 The Florey Institute of Neuroscience and Mental Health, 30 Royal Parade, The University of Melbourne, Parkville, 3052 Victoria Australia; 4 Department of Biochemistry and Biophysics, Stockholm University, Stockholm, Sweden

## Abstract

G-protein coupled receptors are the largest family of integral membrane proteins found within the human genome. They function as receptors and modulators to a wide range of ligands and responses which are crucial for human health. GPCR study, specifically the investigation of structure and interaction to cognate ligands, is of high priority. Limitations for structural study can be traced in part, to obtaining suitable quantities of recombinant protein. We sought to address the limitations of traditional recombinant technologies by utilising an *Escherichia coli* based cell-free protein synthesis (CFPS) approach for production of a thermostable neurotensin receptor 1 (en2NTS_1_). Initial results were promising, with a high amount (up to 2 mg/mL) of en2NTS_1_ produced, that had attained correct secondary structure. Meanwhile, concurrent experiments indicated that CFPS produced en2NTS_1_ showed non-competitive binding to the peptide ligand neurotensin8–13 when compared to *E. coli* produced en2NTS_1_. ^1^H-^13^C HMQC SOFAST NMR spectra were indicative of disrupted tertiary structure for CFPS produced ^13^CH_3_-methionine labelled en2NTS_1_. The results obtained, indicate CFPS produced en2NTS_1_ is not forming a discrete tertiary structure and that further development of the CFPS technique needs to be carried out.

## Introduction

G-protein coupled receptors (GPCRs) are a large family of integral membrane proteins of approximately 800 members in humans^1, 2^. They constitute the largest group of cell-surface proteins in the human genome and are sorted into four families based on characteristic protein sequences and structural similarity to their prototypical namesake^1^: the rhodopsin family (Class-A), the secretin and adhesion family (Class-B), the glutamate family (Class-C) and the Frizzled/Smoothened family (Class-F)^1, 3^. As membrane proteins, GPCRs are the target of many different stimuli that affect a diverse range of responses. Many GPCRs are known to have broad effects on health^4^, and therefore, the large number of GPCRs in the human genome may allude to high numbers of possible pharmacological targets for treatment of many ailments. Notably, it has been estimated that 30–40% of all currently marketed pharmaceuticals directly target GPCRs^5^. As a result, the investigation of GPCR structure and function is of high importance.

The characteristic feature shared between all GPCRs is that they possess a transmembrane (TM) domain composed of seven predominantly α-helical transmembrane passes (7TM)^2^. The 7TM helices are arranged in a serpentine pattern in relation to the lipid bilayer and all GPCRs exhibit the same orientation with respect to their insertion into the lipid bilayer: the N-terminus is exposed to the extracellular face of the lipid bilayer and the C-terminal tail projecting into the intracellular space. The individual TM helices are connected by a series of extracellular and intracellular loops. To date, there have been greater than 115 different GPCR structures, of varying conformational states, deposited into the PDB. Most of the successfully obtained GPCR structures have been determined from protein that is produced by recombinant means. The most common expression organisms used for production of GPCRs include insect cells, *Spodoptera frugiperda* or *Trichopulsia ni* and the yeast, *Pichia pastoris*. The main attribute for expression of GPCRs using these organisms, is that they are processed through a similar secretory pathway as in mammalian hosts. Specifically they allow facilitation of receptor folding pathways, protein processing and disulphide bond formation. Additionally, post-translational modifications (PTMs) such as glycosylation may occur, albeit differing in form depending on expression host.

Alternative production techniques for GPCRs include *Escherichia coli* expression or cell-free protein synthesis (CFPS). Expression in *E. coli* is a proven method for expression of some native or mutant GPCRs in a properly folded form that exhibit native ligand binding. Examples include A_2A_R^6^, β_1_AR^7^, CXCR1^8^ and NTS_1_^9, 10^. Meanwhile CFPS of folded and functionally active GPCRs has only been successful on a few receptors such as the β_2_AR^11^, H_1_R^12^ and dopamine D_2_R^13^. Despite limited success, CFPS is a highly sought after technology to produce GPCRs due to the relatively low cost of materials and high yields of protein production. Additionally, CFPS allows the user to directly manipulate and also include unnatural components to the synthesis reaction. The substitution of isotopically labelled or unnatural amino acids makes CFPS a very attractive alternative to traditional recombinant expression. However, expression of membrane proteins by CFPS is challenging, as expression is carried out with minimal or negligible regulation of protein folding. Therefore the operator must supply an environment suitable for solubilisation of the nascent protein or include a refolding step following expression.

Described here is the investigation to assess the functionality and structure of an engineered thermostabilised neurotensin receptor 1 (en2NTS_1_)^14^, produced by CFPS. The work presented demonstrates the capability of CFPS to produce high levels of GPCR protein. The system allowed significant manipulation, by addition of various detergents and replacement of unlabelled methionine with ^13^C-methyl-methionine for eventual studies of GPCR structure and dynamics by NMR spectroscopy. However ligand binding assays revealed that CFPS produced malE-en2NTS_1_ showed a propensity to bind non-competitively to the peptide ligand neurotensin8–13 (NT_8–13_). Additionally, NMR studies highlighted that CFPS produced en2NTS_1_ displayed a non-native tertiary structure, compared to *E. coli* produced en2NTS_1_, consistent with CFPS producing a poorly folded, non-functional receptor.

## Results

### en2NTS_1_ cloning

**Figure 1:**
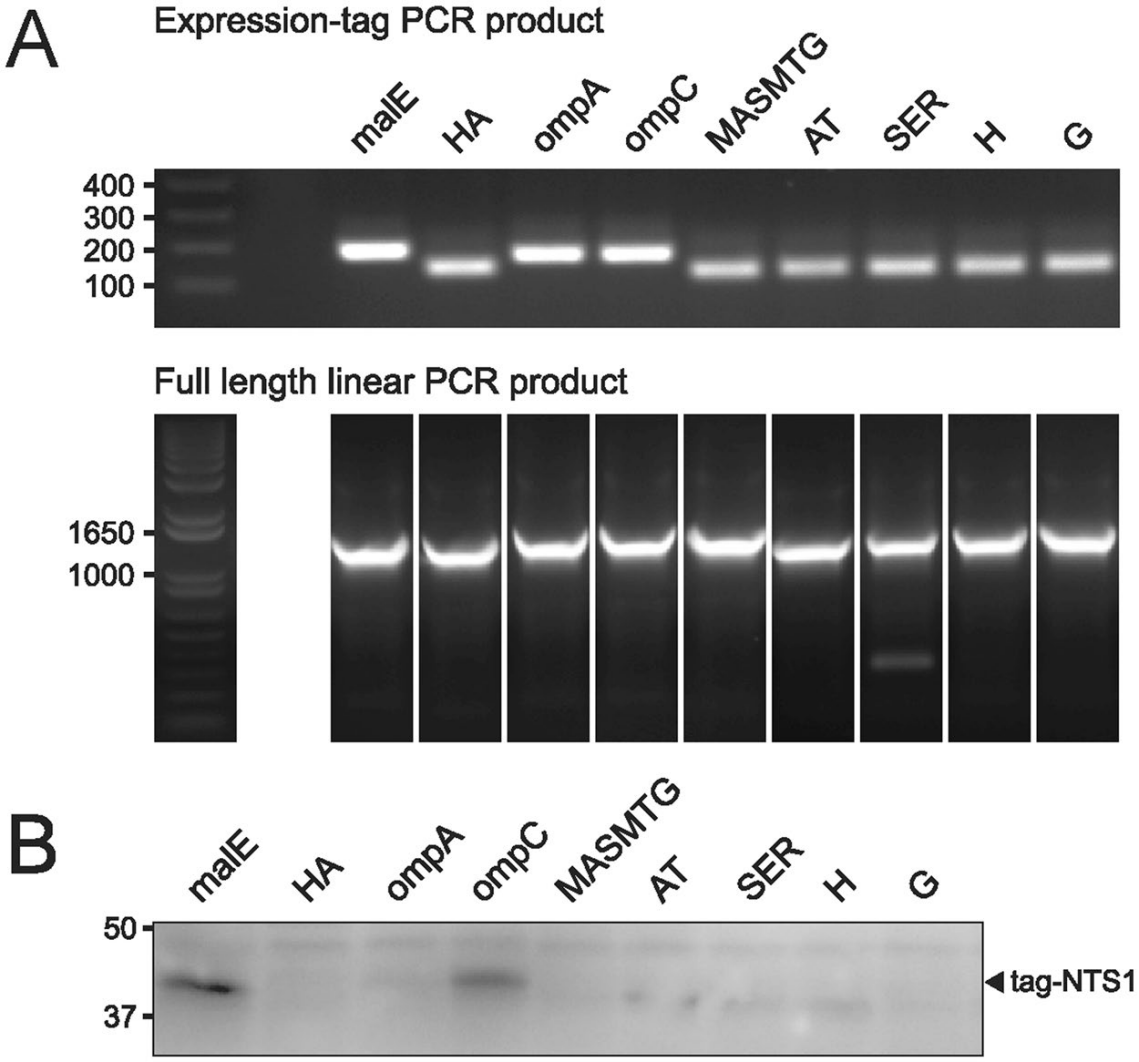
Overlap PCR of linear PCR expression templates and analytical scale CFPS test expression. (**A**) The various expression tags, containing all necessary regulatory elements were generated by an initial PCR. The expression tag, along with the PCR product of en2NTS_1_ was used to generate the full-length PCR product with the all necessary regulatory elements required for *in vitro* expression. (**B**) Expression was assessed by immunoblot against the His-tag found at the C-terminus of en2NTS_1_. The malE-signal sequence was identified as the best candidate for expression by CFPS.

The first requirement for establishing CFPS production of en2NTS_1_ was constructing the necessary template for protein synthesis. Based on previous reports which state that reducing mRNA fold at the 5′- end of coding mRNA can lead to optimal likelihood of translation initiation and protein expression^15^, we sought to create several N-terminally tagged-based en2NTS_1_ variants. Creation of the tagged-en2NTS_1_ variants utilised an overlap PCR approach (Fig. 1A) (Fig. S1 in Supporting Information), whereby nine fusion partners were chosen based on their reported expression enhancement of both soluble and membrane proteins by CFPS^16–18^. The impact of the different expression tags on protein synthesis was performed by analytical scale CFPS expression in the absence of surfactants (precipitate mode CFPS: P-CFPS) so as to allow easy isolation of the product by centrifugation. Expression was observed for most of the N-terminal tags tested, despite an overall lower expression for the linear templates compared to an intact plasmid. Despite the low expression, two signal sequence based tags obtained from malE and ompC promoted sufficient expression, with the 26-residue malE tag producing approximately 47% more product than ompC tagged en2NTS_1_ (Fig. 1B). Thus malE-en2NTS_1_ cloned into pETMCSI (a kind gift from K. Ozawa and G. Otting) was therefore used for continued study.

### malE-en2NTS_1_ expression optimisation and purification

**Figure 2:**
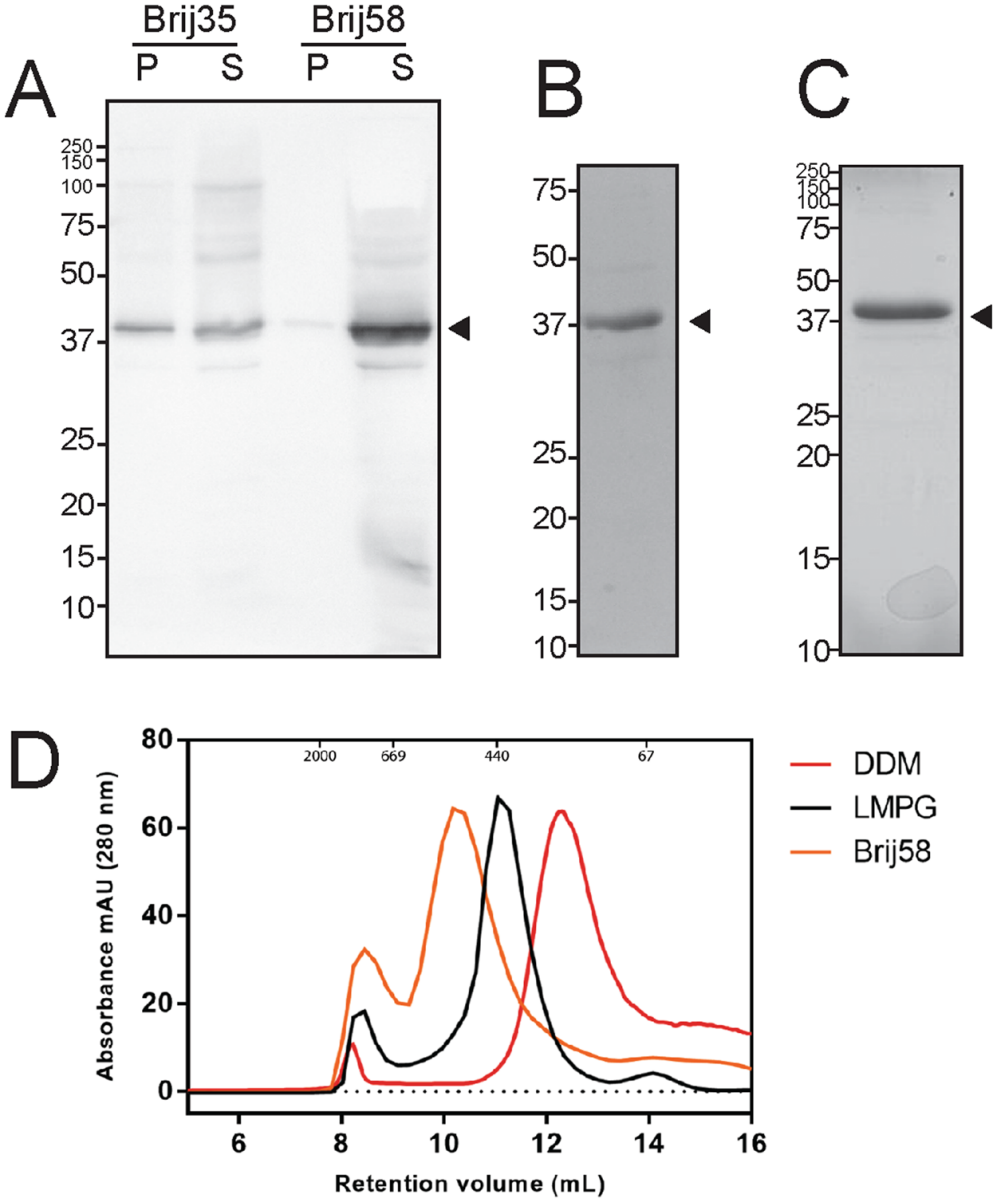
Expression and purification of malE-enNTS_1_. malE-en2NTS_1_ could be expressed in either D-CFPS or P-CFPS mode. (**A**) Anti-His immunoblot highlighting better solubility of malE-en2NTS_1_ in Brij58 compared with Brij35 during D-CFPS mode with near complete solubilisation in the soluble (S) fraction and very little seen in the insoluble pellet (P). (**B** and **C**) Coomassie stained SDS-PAGE of Ni-IMAC purified malE-en2NTS_1_ obtained from D-CFPS and P-CFPS modes respectively. Arrows indicate malE-en2NTS_1_. (**D**) Size-exclusion profiles for malE-en2NTS_1_ in three solubilising detergents, DDM, LMPG and Brij58.

Further optimisation of malE-en2NTS_1_ expression was carried out by testing varying Mg^2+^ concentrations, expression in the presence and absence of detergent and determining the relative yield of protein from CFPS. Titration with Mg^2+^ showed that optimal expression could be obtained at a concentration of 17 mM. Expression could be carried out in the presence of Brij35 (0.1% w/v) and Brij58 (1.5% w/v), with noticeably better expression in Brij58 because a large proportion of malE-en2NTS_1_ was insoluble with Brij35 addition (Fig. 2A). Notably, addition of Triton X-100, dodecylphosphocholine (DPC), decyl maltoside (DM) or dodecyl maltoside (DDM), resulted in no malE-en2NTS_1_ expression. A comparison between detergent mode-CFPS (D-CFPS) using Brij58 and P-CFPS showed P-CFPS generated higher levels of synthesised protein. Overall yields by D-CFPS and P-CFPS were 0.5–1 mg mL^−1^ and 1.5–2 mg mL^−1^ respectively. For malE-en2NTS_1_ expressed in the P-CFPS mode, complete solubilisation of the precipitate was successfully carried out using 21 mM (1% w/v) 1-myristoyl-2-hydroxy-*sn*-glycero-3-phospho-(1′-*rac*-glycerol) (LMPG) in nickel equilibration buffer.

Purification utilised Ni-IMAC to produce a product that was ≥ 90% pure for both D-CFPS and P-CFPS derived malE-en2NTS_1_ (Fig. 2B,C respectively). All samples were purified in either Brij58 (D-CFPS derived), LMPG (P-CFPS derived) or DDM (buffer exchanged from D-CFPS or P-CFPS derived malE-en2NTS_1_). Unfortunately malE-en2NTS_1_ was unable to be detergent exchanged into DM. Several attempts were carried out, however all attempts resulted in precipitation of malE-en2NTS_1_. The precipitated material was determined to be malE-en2NTS_1_ by SDS-PAGE (data not shown). For malE-en2NTS_1_ purified in either DDM, LMPG or Brij58, size-exclusion profiles show monodisperse profiles (Fig. 2D). The varying elution volumes were predicted to be due to the different micellar properties of the solubilising detergents (Fig. S2 in Supporting Information).

### Circular Dichroism and secondary structure

**Figure 3:**
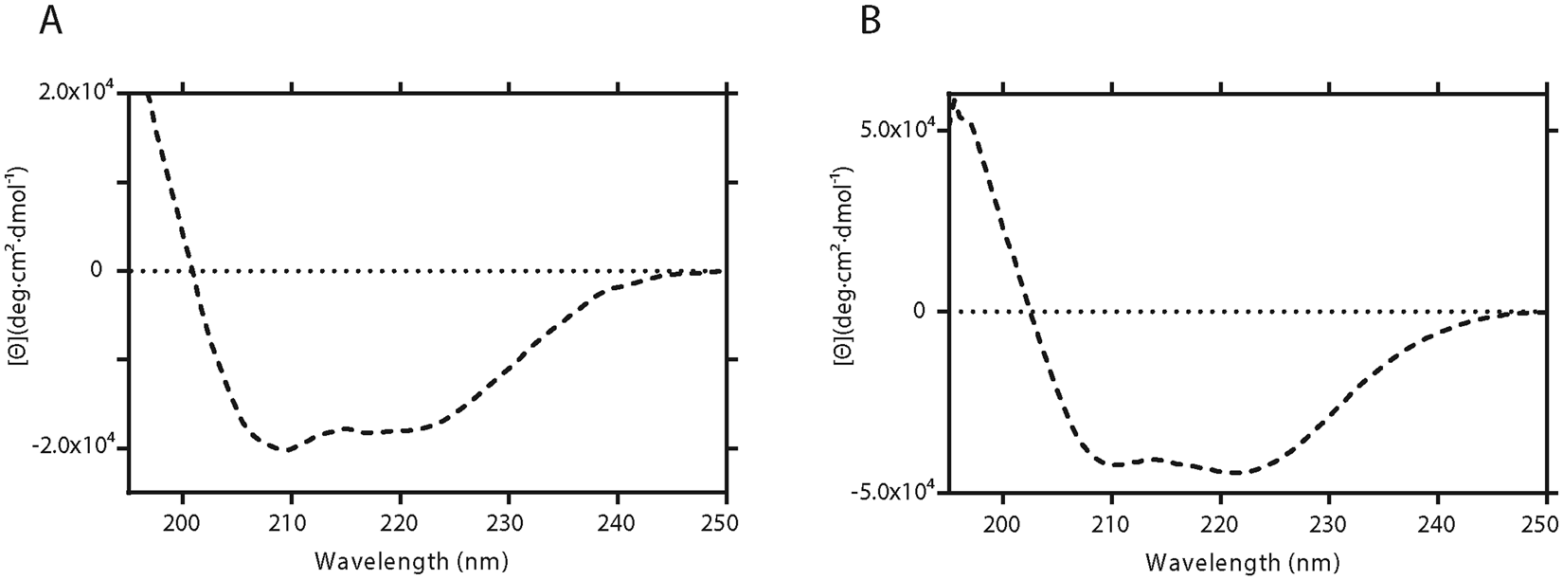
Circular dichroism spectra of malE-en2NTS_1_ and *E. coli* produced en2NTS_1_. Data are represented as mean residue ellipticity for (**A**). CFPS produced malE-en2NTS_1_ and (**B**). *E. coli* produced en2NTS_1_, where both show a profile exhibiting α-helical like structure, as visualised by a pronounced local minima at 208 nm and 222 nm.

Circular dichroism (CD) was carried out to determine the estimated secondary structure of malE-en2NTS_1_. The samples used for CD were derived either from D-CFPS and P-CFPS expressed malE-en2NTS_1_. In both cases however, detergent exchange to DDM took place during the Ni-IMAC step and DDM was maintained during subsequent SEC and CD steps. The far-UV wavelength CD profile of malE-en2NTS_1_, which includes the 26-residue malE tag, displayed a content of secondary structure consistent with that of a folded GPCR (Fig. 3A). This was broadly similar to the control CD profile obtained for *E. coli* produced en2NTS_1_, however the mean residue ellipticity is lower for the cell-free produced material (Fig. 3B). The two double minima present at 208 and 222 nm were suggestive of a protein with a high propensity of α-helix. The reconstituted data were fitted best with the algorithms CDSSTR and CONTILL, producing the lowest nRMSD values and therefore a more accurate fit of the acquired data^19^ (Table 1). When analysed, the overall secondary structure composition calculated by CDpro produced an estimation of α-helix similar to data obtained from a second program, STRIDE^20^. The calculated α-helical percentage (58–60%) and the limited amount of β-strand (8–13%) was similar to the calculated CD profile for *E. coli* produced en2NTS_1_ as well as the amount determined for the NTS_1_-OGG7 crystal structure (OGG7-ΔIC3, PDB: 4BV0), to which both malE-en2NTS_1_ and en2NTS_1_ share many of the same stabilising mutations^10^ (Fig. S3 in Supporting Information). This analysis is suggestive of malE-en2NTS_1_ assuming a structure that is consistent with correct secondary fold, however the lower value of the mean residue ellipticity is consistent with poor packing of the α-helices to form the correct tertiary structure^21^. Clearly, in the absence of the *E. coli* expressed en2NTS1 such an analysis is not possible.

**Table 1:** Percentage composition of secondary structure for malE-en2NTS_1_ and *E. coli* produced en2NTS_1_ compared to structural data using pdb data inputted into STRIDE.

Data sets	CD data/CDpro output Algorithm: CDSSTR	CD data/CDpro output Algorithm: CONTIN-LL	CD data/CDpro output Algorithm: CDSSTR	CD data/CDpro output Algorithm: CONTIN-LL	STRIDE^a^ analysis of NTS_1_-OGG7
	malE-en2NTS_1_	malE-en2NTS_1_	en2NTS_1_	en2NTS_1_	NTS_1_-OGG7 (4BV0)
α-helix total (%)	60.5	58.6	56.1	69.3	63.1
β-strand total (%)	13	8.4	16	1	2.7
Turns (%)	13.8	23.2	13.8	4.4	7.2
Unordered (%)	10.9	9.7	14.3	25.3	6.4
Unknown (%)	—	—		—	20.6
nRMSD	0.03	0.106	0.08	0.224	—

^a^STRIDE calculations were based on the NTS_1_-OGG7 structure with the pdb accession code 4BV0. 4BV0 is a stable NTS_1_ variant lacking most of ICL3, and most of the N- and C-terminus. In addition only 297 residues of NTS_1_-OGG7 were identifiable compared to 374 residues of malE-en2NTS_1_. Therefore, for this analysis, secondary structure content was calculated for 374 residues resulting in missing information for 20.6% of the protein.

### Streptavidin and His_6_-tag pulldown assays of malE-en2NTS_1_

**Figure 4:**
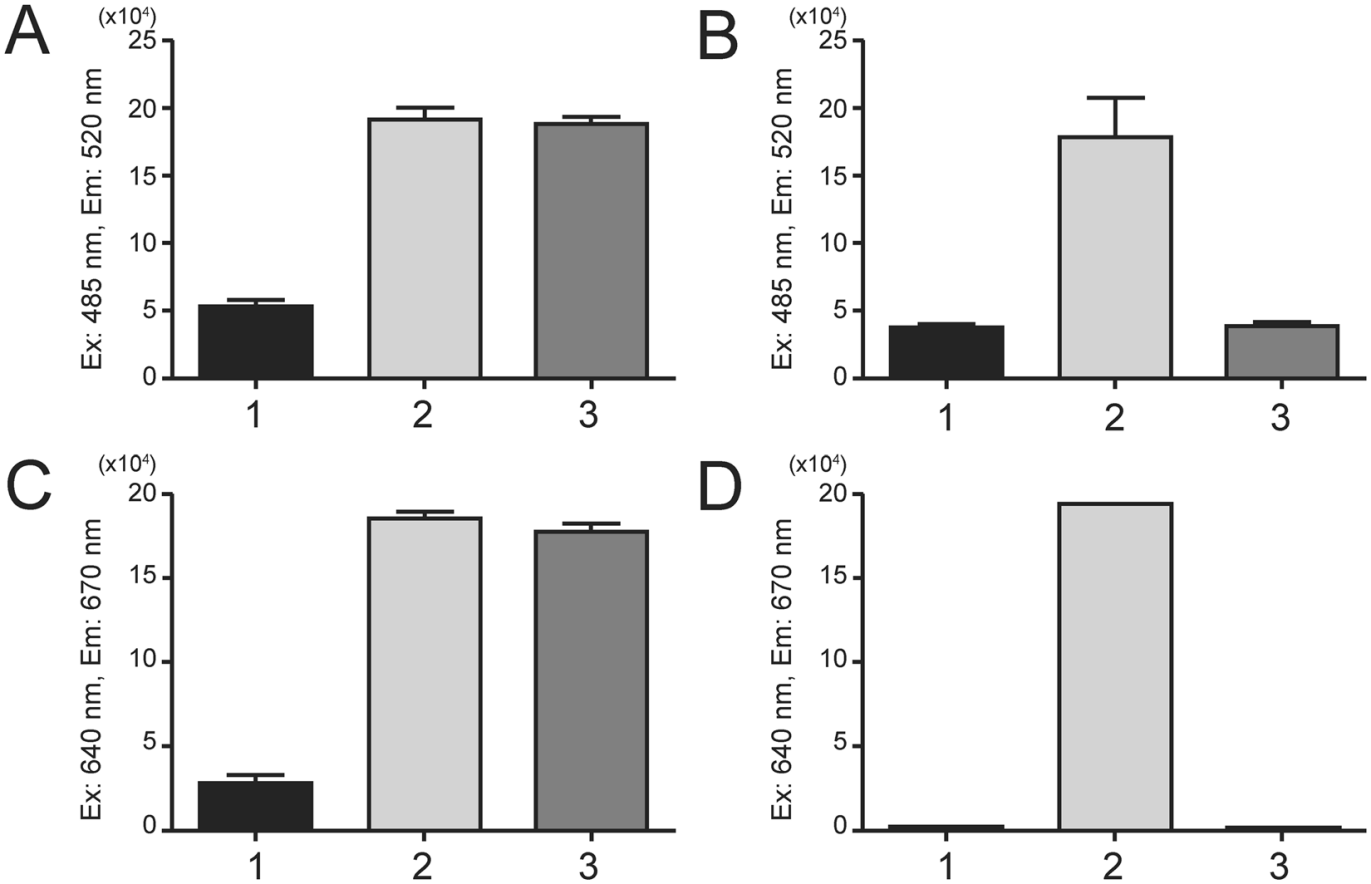
Streptavidin and His-tag pull down assay of CFPS malE-en2NTS_1_ and *E. coli* expressed en2NTS_1_. Binding assay, testing capability of binding to Biotin-NT using Streptavidin dynabeads in combination with (**A**). CFPS produced malE-en2NTS_1_ and (**B**). The *E. coli* expressed en2NTS_1_ control. Each graph reads 1. Receptor alone. 2. Biotin-NT plus receptor. 3. Biotin NT, receptor and unlabelled NT_8–13_ competitor. Binding assay using His-tag isolation dynabeads in combination with (**C**). CFPS produced malE-en2NTS_1_ (**D**). *E. coli* expressed en2NTS_1_. Each graph reads 1. Receptor alone. 2. A647-NT_8–13_ plus receptor. 3. A647-NT_8–13_, receptor and NT_8–13_ competitor. Data is presented as triplicate experiments ± SEM.

Automated streptavidin binding experiments were carried out using the kingfisher particle processor. Streptavidin-coated dynabeads were used to capture biotinylated NT, which was subsequently used to capture CFPS produced malE-en2NTS_1_ (Fig. 4A). *E. coli* expressed en2NTS_1_ (Fig. S3) served as a control for expected interaction of the receptors with the NT_8–13_ ligand (Fig. 4B). The experimental set up included three conditions; 1: Receptor alone, 2: biotinylated-NT and receptor and 3: biotinylated-NT, receptor and unlabelled NT_8–13_ competitor. No binding of malE-en2NTS_1_ or of en2NTS_1_ was detected to the streptavidin dynabeads in the absence of bound biotin-NT. Addition of biotin-NT promoted binding of malE-en2NTS_1_ and en2NTS_1_ to the beads. Addition of competing unlabelled NT_8–13_ caused a reduced level of bound en2NTS_1_, but did not change the level of bound malE-en2NTS_1_ to the biotin-NT-coated streptavidin dynabeads. For malE-en2NTS_1_, samples obtained following Ni-IMAC in the detergents Brij58, LMPG or DDM produced the same result, as did samples purified by SEC in DDM (data not shown).

In light of the streptavidin pulldown experiments, an alternative approach was attempted. malE-en2NTS_1_ was designed with a His_10_-tag at its C-terminus, a trait that is exploited during its purification. With this in mind, the His-tag was used to reverse the original streptavidin method for detection of possible binding of NT_8–13_. By coupling the malE-en2NTS_1_ receptor to His-tag isolation dynabeads, detection of binding can be made through Alexa647 fluorescently labelled NT^14^. Once again, malE-en2NTS_1_ binding experiments could be compared in a similar manner to the *E. coli* expressed en2NTS_1_ control which can also be captured via its His-tag. Results from several experiments however produced similar results to the previous streptavidin assay (Fig. 4C,D). In the absence of bound receptor, A647-NT_8–13_ showed no interaction with the His-tag isolation dynabeads. Binding of both receptors to the His-tag isolation dynabeads, promoted binding of A647-NT_8–13_. The co-incubation of excess unlabelled NT_8–13_ as a competitor resulted in no dissociation of A647-NT_8–13_ from CFPS produced malE-en2NTS_1_, whereas complete competition of A647-NT_8–13_ binding was observed from *E. coli* produced en2NTS_1_ immobilised beads (Fig. 4D).

### ^1^H-^13^C SOFAST HMQC NMR of en2NTS_1_

^13^CH_3_- methionine labelled malE-en2NTS_1_ was purified from either P-CFPS or D-CFPS derived sources in DDM and Brij58 detergents respectively. malE-en2NTS_1_ in the NT agonist bound state was recorded by adding excess NT_8–13_ in a ratio of 10:2 NT_8–13_:receptor (100 µM:20 µM). Following NMR acquisition, the sample was checked for any precipitation by visual inspection of the sample. Spectra were obtained for malE-en2NTS_1_ (apo and 100 μM NT_8–13_) in the presence of 1 mM DDM or 1 mM Brij58.

**Figure 5:**
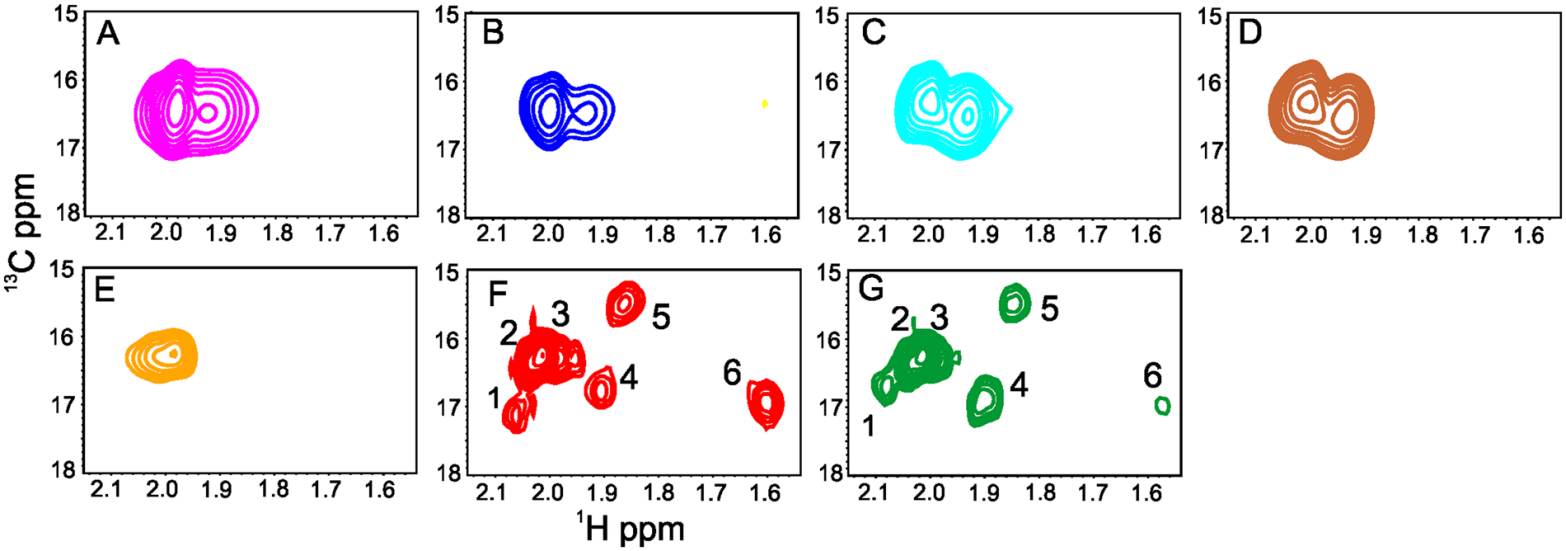
^1^H-^13^C SOFAST-HMQC spectra of ^13^CH_3_-methionine labelled malE-en2NTS_1_ and en2NTS_1_. (**A**). malE-en2NTS_1_ (apo) in DDM (**B**). malE-en2NTS_1_ (100 μM NT_8–13_) in DDM. (**C**) malE-en2NTS_1_ (apo) in Brij58. (**D**) malE-en2NTS_1_ (100 μM NT_8–13_) in Brij58. (**E**) malE-en2NTS_1_ (50 μM NT_8–13_) in POPC nanodiscs. (**F**) en2NTS_1_ in DDM. (**G**) en2NTS_1_ (500 μM NT_8–13_) in DDM.

MalE-en2NTS1 has three methionines in the malE tag and nine within the transmembrane domain (Figs S3 and S4 in Supporting Information). However, the spectra for apo malE-en2NTS_1_ in both detergents displayed two broad signals (Fig. 5A,C) compared to *E. coli* derived en2NTS_1_ in DDM, which showed clearly defined resonances for at least six of nine possible methionine signals (Fig. 5F). Alternatively, addition of NT_8–13_ to CFPS-derived malE-en2NTS_1_ promoted no change in the size of the resonance, nor was there any noticeable change in chemical shift (Fig. 5B,D) whereas addition of NT_8–13_ to *E. coli* derived en2NTS_1_ resulted in chemical shift and/or line width changes for four of the resonances (Fig. 5G). Of particular note was the observed shape of both P-CFPS and D-CFPS derived forms of malE-en2NTS_1_, where a comparison of resonances derived from samples purified from D-CFPS and P-CFPS malE-en2NTS_1_ (Fig. 5A,C) indicate, that regardless of the CFPS method for malE-en2NTS_1_ production, the final purified forms are displaying similar properties.

MalE-en2NTS_1_ labelled with ^13^CH_3_- methionine was expressed in the presence of POPC nanodiscs. NMR was carried out on Ni-IMAC purified malE-en2NTS_1_, in the same manner used for detergent purified malE-en2NTS_1_. From the spectra, only a large single resonance was detectable, although the line-width of this single resonance compared to DDM/Brij58 solubilised malE-en2NTS_1_ appeared narrower (Fig. 5E). Comparison to the spectra obtained for en2NTS_1_ (Fig. 5F) show none of the defined resonances expected of a protein adopting a distinct tertiary structure.

Following NMR acquisition, the sample was applied to SEC to identify whether the nanodisc fraction still maintained the expected elution profile. Nanodiscs that utilise the MSP1D1 scaffold have a defined diameter of ~100 Å or 10 nm without embedded membrane protein and may be slightly larger with protein embedded^22, 23^. When compared to protein standards, nanodiscs of correct size eluted at the same volume as catalase, due to their shared Stokes diameter. For the malE-en2NTS_1_/POPC complex, the elution did not overlap with the expected elution for catalase, instead eluting in the predetermined void-volume, which is indicative of misfolded protein (Fig. S5 in Supporting Information). From the SDS-PAGE of the fractions obtained by SEC, it can be concluded that the protein was associated with the MSP1D1 scaffold, however the “complexes” were very large, suggesting that malE-en2NTS_1_ had possibly promoted disruption of the disc structure.

It should be noted here, that we have made attempts to incorporate purified malE-en2NTS_1_ into nanodiscs and liposomes composed of POPC, DMPC or *E. coli* total lipid extract. Regrettably all attempts to reconstitute malE-en2NTs_1_ resulted in protein destabilisation and protein precipitation following detergent removal by use of biobeads or dialysis. For nanodisc reconstitution, changing the incubation time, ratios of malE-en2NTS1, lipid and MSP1D1 scaffold protein did not prevent protein precipitation.

## Discussion

G-protein coupled receptors are a diverse set of membrane proteins that respond to an equally diverse range of external stimuli. The many roles that GPCRs play in human physiology highlights their importance and therefore their continued study. Over the years, techniques to produce GPCRs recombinantly have led to an expansion in the details surrounding their structure and function. GPCRs have typically been generated via insect (*S. frugiperda or T. ni)* or yeast (*P. pastoris)* cell expression, which allows for processing by the secretory pathway^24, 25^. Alternate expression methodologies include that of *E. coli* expression, by both membrane integration^10, 26, 27^ or by inclusion body formation^8^. Cell-free protein synthesis offers a fourth alternative for the production of GPCRs^11–13^, with exciting prospects for introducing unnatural amino acids and for allowing direct access to solubilising detergent, all of which formed the basis for this work.

Outlined here is the design, production, purification and initial characterisation of cell-free produced malE-en2NTS_1_, a thermostable NTS_1_ receptor^14^. This receptor was chosen for its proven ability for expression in *E. coli* cell systems (the basis for the CFPS extract used here), high stability in short chained detergents and at elevated temperature and more recently a similar variant was successfully crystallised and its structure determined^10^.

Work proceeded first with en2NTS_1_ by designing suitable N-terminal tags for the quick generation of PCR templates. This methodology allows for the production of multiple templates for rapid determination of expression^16–18, 28, 29^. Nine tags were chosen with PCR templates for all tags generated in one day. CFPS proceeded the following day and determination of protein expression on the third day. Ultimately CFPS of the malE-tagged en2NTS_1_ demonstrated that it was the optimal construct, with high levels of protein production in the 1.5–2 mg/mL range.

Introduction of individual detergents into the CFPS reactions was shown to promote or perturb malE-en2NTS_1_ solubility. Of the detergents tested, only Brij35 and Brij58 resulted in soluble protein expression, with Brij58 promoting complete solubilisation (Fig. 2A). Alternatively samples of malE-en2NTS_1_ derived from CFPS reactions in the P-CFPS mode were able to be solubilised into a refolding solution containing 1% (w/v) LMPG. The properties of malE-en2NTS_1_ detergent solubilisation, during D-CFPS and following P-CFPS, show consistencies with previously published work^30^. Surprisingly however, problems arose during detergent exchange steps, where either Brij58 or LMPG were exchanged to DM. In all instances of detergent exchange to DM, protein destabilisation was observed, culminating in overt protein precipitation. Comparatively, detergent exchange into DDM resulted in no precipitation. malE-en2NTS_1_ destabilisation in DM was not expected, considering the reported stability of en2NTS_1_ in shorter and thus more destabilising detergents, such as octyl-glucoside (OG)^10, 14^. However, the observed differences in stability were noted to possibly be in part due to the slight differences in protein sequence between malE-en2NTS_1_ and en2NTS_1_, such as the addition of the malE-tag.

Investigation of secondary structure by CD showed that malE-en2NTS_1_ possessed the expected α-helical elements of a folded GPCR and matched the estimated secondary structure elements of the *E. coli* produced en2NTS1 and the solved NTS1-OGG7 structure. However, despite the optimistic structural characteristics of malE-en2NTS_1_, the protein bound NT_8–13_ non-specifically using either NT immobilised streptavidin beads or receptor immobilised His-tag isolation dynabeads. The ligand binding experiments of malE-en2NTS_1_ could once again be compared with active/folded en2NTS_1_ isolated from *E. coli*. These data indicate that despite having consistent secondary structure elements, it possibly had not adopted a suitably discrete tertiary structure, which prevented the correct ligand association and subsequent competition. ^1^H-^13^C HMQC SOFAST NMR experiments showed quite conclusively that malE-en2NTS_1_ does not exhibit the same proper tertiary structure and therefore was not behaving in accordance with a correctly folded receptor. Comparisons to the *E. coli* produced en2NTS_1_, which does bind peptide as expected and exhibited defined tertiary structure by NMR, is further evidence for the lack of defined tertiary structure for CFPS synthesised malE-en2NTS_1_.

The noted stability of NTS_1_ engineered variants expressed in *E. coli* ^14^, in comparison to CFPS produced malE-en2NTS_1_, may indicate that a number of structural features need to be attained prior to their purification; structural features that are not easily attainable in a CF system, in the absence of a lipid bilayer and membrane insertion machinery. When expressed in *E. coli*, en2NTS_1_ is shuffled into the lipid bilayer directed by the N-terminally fused MBP. Once exposed to the membrane folding pathway at the SecYEG-translocon (SecYEG), several factors ensure that packing of the α-helical TM segments occurs correctly and that interhelical contacts are established^31^. Without the assistance of the SecYEG and associated protein chaperones, membrane proteins are thought to rapidly collapse into non-native conformers^32^. If this is the case, then refolding of malE-en2NTS_1_ by employing a mild LMPG solubilisation or directly into a detergent may not be sufficient to reverse any structure assumed during *in vitro* translation.

It is also possible that thermal stability of en2NTS_1_ gained through successive rounds of directed evolution does not increase the propensity to adopt the correct fold, unassisted, from an unfolded/semi-folded state. Likewise, malE-en2NTS_1_ derived from CFPS did not respond in a manner expected of a functional receptor. While most native GPCRs would unfold in conditions where short chain detergents are present, en2NTS_1_ produced in *E. coli* does not. Thus, the structural stability of bacterially produced en2NTS_1_ in detergents such as DDM, DM or OG is contingent on the receptor first being properly folded in the *E. coli* membrane environment conducible to receptor folding, prior to detergent solubilisation.

One approach to supplying nascent malE-en2NTS_1_ with a lipid bilayer was undertaken by supplementing the CFPS reaction with preformed nanodiscs^33^ comprised of a POPC core and MSP1D1 scaffolding. Recently there have been several reports of direct CF expression of GPCRs into lipid nanodiscs^34, 35^. These embedded receptors were also reported to exhibit activity, therefore offering a new method for obtaining this class of receptor in a biologically active form. Successful CFPS expression of malE-en2NTS_1_ in the presence of nanodiscs and their subsequent purification also hinted at the possibility of this receptor being embedded within the nanodisc lipid core. As before, the ^1^H-^13^C HMQC SOFAST acquisition was unable to detect individual dispersed resonances exhibited by *E. coli* produced en2NTS_1_. Unfortunately, a comparison of malE-en2NTS_1_ associated with the POPC nanodiscs showed a single resonance dispersal pattern similar to that of detergent solubilised malE-en2NTS_1_. Following NMR acquisition, the malE-en2NTS_1_/nanodisc sample was applied to gel filtration, which confirmed the aberrant NMR results were most likely due to misfold and collapse of the preformed nanodisc structure.

While the goals we set out to achieve were not met, a number of useful procedures could be implemented as generic means for assessing membrane proteins produced by CFPS. Notably the use of competition binding assays to identify whether interactions with target substrates/ligands are correct. Alternatively, if a suitable ligand is unavailable, isotopic labelling during CFPS, such as ^13^CH_3_-methionine, can be used for an economic and rapid determination of protein fold.

For malE-en2NTS_1_, it seems likely that it would not be possible to obtain a functional form of this receptor when derived from CFPS, unless a more radical approach to its production is undertaken. This would require malE-en2NTS_1_ expression to be undertaken in the presence of a fully functional membrane translocon. This has been trialled before with microsomes or through trials of membrane incorporation with components of SecYEG and affiliated proteins^36^. In another test case, GPCRs have been incorporated into giant unilamellar vesicles originating from endoplasmic reticulum^37^. Alternatively, incorporation of *E. coli* microsomes derived from inner membrane vesicles (IMV) could possibly serve as a substitute to ER derived membranes. One study has shown *E. coli* IMVs are capable of incorporating two *E. coli* transporters, mannitol permease (MtlA) and the tetracycline pump (TetA)^38^. Of the total protein expressed, MtlA and TetA were successfully incorporated into the microsome bilayer at 38% (130 µg/mL) and 66% (570 µg/mL) respectively^38^. The yields for CFPS membrane incorporation are currently medium-low for these types of systems, owing to limitations in incorporation rates and total membrane area. Whether non-*E. coli* derived proteins could be incorporated into an *E. coli* based CFPS system with *E. coli* derived IMVs, is a matter for future investigation. However this may offer a potential method for production of folded and functional *in vitro* synthesised membrane protein and GPCRs as a whole.

## Methods

### enNTS1 plasmid construction

PCR was used to generate several different linear fused-gene DNA templates of enNTS1, each with their own unique expression tag for quick determination of levels of expression by CFPS (Fig. S1 in Supporting Information). The constructs were designed to have en2NTS_1_ preceded by an N-terminal expression tag followed by a 3C-protease site and succeeded by a C-terminal His_10_-tag. The following expression tags were selected based on previously reported accounts: the signal sequences of *E. coli* ompA, ompC, malE^16^, an HA-tag (Roche pIVEX2.6d vector), truncated T7-tag (MASMTG)^18^, and the systematically designed expression tags AT, SER, H and G^17^. Once determination of optimal en2NTS_1_ expression was made, the thermostabilised en2NTS_1_ gene was subcloned using the NdeI and EcoRI restriction sites into pETMCSI. Generation of the malE-en2NTS_1_ fusion, was generated by a combination of overlap PCR, using the primers P1, P8, P9, P14 and P15 (Table S1 in Supporting Information).

### Continuous exchange cell-free protein synthesis

Preparation of *E. coli* BL21(DE3) S30 extracts as well as the CFPS reaction was performed as described by Apponyi *et al*.^39^ with minor changes described below. *E. coli* S30 extract production was prepared using the BL21(DE3) Rosetta strain. These latter cells were grown using the YTPG growth medium described in Choi *et al*.^40^. The cell culture OD_600_ was monitored during the growth phase. Once the OD_600_ reached 0.85, the culture was induced with 1 mM IPTG allowing production of T7RNAP to take place, thus preventing separate expression and purification of this vital component.

Each CFPS reaction was composed of 0.8 mM rNTPs (CTP, UTP, GTP), 1.2 mM ATP, 55 mM HEPES pH 7.2, 68 µM Folinic acid, 0.64 mM 3′,5′-cyclic AMP, 1 mM DTT, 27.5 mM Ammonium acetate, 1 mM amino acids, 80 mM creatine phosphate, 290 mM Potassium acetate, 16–17 mM Magnesium acetate, 7.7 mM Sodium azide, 1x protease inhibitor, 1 mM six amino acid mix (RCWMDE)^30, 41^, 0.3 U RNase inhibitor, 250 µg mL^−1^ creatine kinase, 175 µg mL^−1^ *E. coli* total tRNA, 20–40% (v/v) S30 extract, 16 µg mL^−1^ DNA plasmid template. For reactions requiring use of ^13^CH_3_-methionine, amino acid stocks incorporated 0.5 mM ^13^CH_3_-methionine and 1 mM of the 19 other amino acids.

For continuous exchange CFPS the reaction chamber was prepared as described in Apponyi *et al*.^39^. The reaction chamber was separated from a feeder chamber which supplies new substrate for continued protein production. This was achieved by enclosing the contents of the reaction chamber (S30 extract, plasmid DNA, RNase inhibitor, creatine kinase and *E. coli* tRNA) in dialysis tubing. The dialysis bag is then placed in a feeder solution which supplies new substrate and also serves to minimise undesirable by-products from the protein synthesis reaction. Reactions were expressed in a 1:14 volume ratio (reaction chamber: feeder chamber volume) and incubated at 30 °C with orbital shaking at 160 rpm for 16 hours. Protein expression was carried out in either the presence or absence of detergent or nanodiscs; D-CFPS, P-CFPS or nanodisc (ND-CFPS) modes respectively.

### Nanodisc production

Nanodisc production and assembly was performed with the MSP1D1 construct according to previously established protocols^42^. For this work, POPC nanodiscs with a diameter of ~100 Å were produced and utilised during CFPS at a concentration of 50 μM.

### Protein purification of protein from cell-free synthesis

#### Precipitate mode CFPS – protein solubilisation

Precipitated malE-en2NTS_1_ produced by P-CFPS was first separated from the overnight reaction mix by centrifugation at 17,000 × g for 10 minutes. The isolated malE-en2NTS_1_ required an initial detergent solubilisation step in 21 mM LMPG in nickel equilibration buffer (20 mM Na_2_HPO_4_-NaH_2_PO_4_ pH 7.5, 500 mM NaCl, 5 mM imidazole pH 7.5). The CFPS produced malE-en2NTS_1_ was allowed to mix for one hour with shaking at room temperature. Once solubilisation was complete, the solution was clarified by centrifugation at 17,000 × g for 10 minutes. The clarified solution was then used for Ni-immobilised metal affinity chromatography (Ni-IMAC).

#### Detergent mode CFPS

Following overnight D-CFPS of malE-en2NTS_1_, the final reaction required solution clarification by centrifugation at 17,000 × g for 10 minutes. The sample solution was then adjusted to match the nickel equilibration buffer by dilution whilst maintaining the minimum CMC of the utilised detergent. This final solution was then used for Ni-IMAC.

#### Chromatography purification

malE-en2NTS_1_ samples were incubated with 2 mL of prequilibrated Ni-sepharose (GE Healthcare) for 1 hour at 4 °C. After collecting the flow through, the Ni-sepharose was washed with 10 column volumes of buffer (20 mM Na_2_HPO_4_-NaH_2_PO_4_ pH 7.5, 500 mM NaCl, 5 mM imidazole pH 7.5, and appropriate detergent). If detergent exchange into DDM (20 mM or 1% (w/v)) was to be incorporated, this would occur during the wash step. Protein was eluted using 5 column volumes of elution buffer (20 mM Na_2_HPO_4_-NaH_2_PO_4_ pH 7.5, 500 mM NaCl, 500 mM imidazole pH 7.5, and appropriate detergent). Detergent concentrations were dependent on the individual CMC of the detergent used.

Ni-IMAC purified malE-en2NTS_1_ was loaded onto a Superdex200 10/300 GL column pre-equilibrated with 20 mM Na_2_HPO_4_-NaH_2_PO_4_ pH 7.5, 150 mM NaCl, and appropriate detergent. The size exclusion step was carried out using an FPLC system (Äkta basic, GE healthcare) with buffer kept at 4 °C at a rate of 0.4 mL/min and detected by absorbance at 280 nm. Fractions of importance were collected and analysed by SDS-PAGE for purity.

### en2NTS_1_ expression and purification from *Escherichia coli*

#### Cloning and expression

The en2NTS_1_ sequence was subcloned into a pQE-30-derived vector. The open reading frame of the modified vector encoded an N-terminal maltose-binding protein signal sequence (MBPss), followed by a His_10_-tag, a maltose binding protein (MBP), a NNNNNNNNNNG linker and a HRV 3 C protease site which were linked via a BamHI restriction site (resulting in additional residues GS) to residue T42 of the receptor. C-terminally T420 of the receptor was linked via a NheI restriction site (resulting in additional residues AS) to an Avi-tag for *in vivo* biotinylation, a HRV 3 C protease site, a GGSGGS linker and a monomeric ultra-stable green fluorescent protein (musGFP)^43^, which is followed by a second His_10_-tag.

Expression and ^13^CH_3_-methionine labelling of the MBP-en2NTS_1_-musGFP fusion protein was carried out in *E. coli* C43(DE3) (Lucigen) following an adapted protocol described by Van Duyne *et al*.^44^.

#### Chromatography purification

Cell pellets were resuspended in 100 mM HEPES, 400 mM NaCl, 20% Glycerol, pH 8 with 1x EDTA free protease inhibitor tablet (Roche), 100 mg Lysozyme, 10 mg DNAse and sonicated on ice. Following sonication 15 mL of DM solution (1.6 g n-decyl-β-D-maltopyranoside, Anagrade (Antrace) dissolved overnight in water) and 15 mL of CHS/CHAPS solution (0.12 g cholesterol hemi succinate (Sigma) and 0.6 g CHAPS-hydrate (Sigma) dissolved overnight in water) were added and the volume was adjusted to 100 mL. The solubilisation mix was left gently rocking for 2 h at 4 °C. Cell debris was removed by centrifugation.

Filtered (45 µm) supernatant was adjusted to 10 mM imidazole, passed over 6 mL of Talon resin, washed with 2 × 25 mL of 25 mM HEPES, 500 mM NaCl, 10% Glycerol, 0.15% DM, 10 mM Imidazole, 0.2 mM PMSF, 8 mM ATP, 10 mM MgCl2, pH 8. Detergent was exchanged to DDM (n-decyl-β-D-maltopyranoside) (Anagrade, Anatrace) by washing with 2 × 25 mL of 25 mM HEPES, 100 mM NaCl, 10% Glycerol, 0.05% DDM, 0.2 mM PMSF, pH 8. The fusion protein was eluted using 15 × 1 mL of 25 mM HEPES, 100 mM NaCl, 10% Glycerol, 0.05% DDM, 350 mM Imidazole, 0.2 mM PMSF, pH 8.

The eluate was concentrated to 1 mL using an Amicon Ultra 15 concentrator with 100 kDa cutoff (Millipore); buffer exchanged using a PD10 desalting column (GE Healthcare) into 25 mM HEPES, 300 mM NaCl, 10% Glycerol, 0.05% DDM, pH 8. Proteolytic cleavage was carried out by adding 100 mM of Na_2_SO_4_, 1 mM TCEP and 30 µL of GST-tagged HRV 3 C protease (96 μM stock produced in house) to the 4 mL PD10 eluate and rocking gently for 16 h at 4 °C. The protease was removed by mixing for 1 h at 4 °C with 2 mL of Glutathione Sepharose 4B (GE Healthcare). The flow-through was collected and the GST resin washed with 15 mL of buffer. GST flow-through and wash were combined, adjusted to 5 mM Imidazole and transferred to a gravity flow column containing 6 mL of Talon resin equilibrated with 2 × 25 mL of 25 mM HEPES, 300 mM NaCl, 10% Glycerol, 0.05% DDM, pH 8. The mixture was rocked for 45 min at 4 °C. The flow-through containing cleaved enNTS1 was collected, the beads washed with 3 × 10 mL of buffer.

The flow-through and washes were combined and concentrated to 450 µL using an Amicon Ultra 15 concentrator with 30 kDa cutoff (Millipore). The concentrate was transferred to an Eppendorf tube and centrifuged in a table-top centrifuge (10000 rpm, 4 °C, 10 min) to separate any aggregated protein. The supernatant was loaded and further purified on a Superdex 200 10/300 Increase column (GE Healthcare) equilibrated with 50 mM potassium phosphate, 100 mM NaCl, 0.02% DDM, pH 7.4.

#### Circular dichroism spectroscopy

Far UV circular dichroism (CD) spectroscopy was carried out to estimate the secondary structure of malE-en2NTS_1_. Measurements were performed on an AVIV Model 410 SF spectropolarimeter (Aviv) equipped with a temperature controlled jacket set at 25 °C. malE-en2NTS_1_ samples were maintained in a minimal salt buffer (20 mM Na_2_HPO_4_-NaH_2_PO_4_ pH 7.5, 50 mM NaCl, 0.3 mM DDM). Wavelength scans were assayed at the far-UV range from 250 to 190 nm at 0.5 nm intervals and in triplicate for averaging. Protein samples (0.1 mg mL^−1^) were housed in a 1-mm light path quartz cuvette (Starna). Data measured from wavelengths 250–190 nm were expressed as mean residue ellipticity [θ] and fitted using the CDpro software^45^ running three algorithms to provide an estimate of the secondary structure composition; SELCON3^46^, CDSSTR^47^ and CONTINLL^48^. All algorithms used the protein reference set SMP56^49^ which includes 43 soluble proteins and 13 membrane proteins for comparison.

#### Ligand binding assay

Ligand binding assays of malE-en2NTS_1_ and en2NTS_1_ were performed on the KingFisher Flex Magnetic Particle Processor (Thermo Scientific) carried out at 4 °C. For malE-en2NTS_1_ pulldown experiments, two different dynabead matrices were used; Streptavidin coupled M-280 dynabeads or His-tag isolation and pulldown dynabeads (Life Technologies). Streptavidin coupled M-280 dynabeads were incubated with biotinylated neurotensin (produced in house via reaction between maleimide-PEG2-Biotin and a cysteine modified NT) for one hour. malE-en2NTS_1_ receptor binding was assessed by binding of a primary mouse Anti-His antibody (1:1000, Cell Signalling Technologies) followed by binding of anti-mouse-Alexa488 antibody (1:5000, Life Technologies). Competition was assessed through incubation of unlabelled NT_8–13._ The mixing protocol was as follows, with washing for 10 minutes between each step: streptavidin dynabeads were incubated with biotinylated NT for one hour, then with malE-en2NTS_1_ or en2NTS_1_ ± competitor NT_8–13_ for another hour, with mouse Anti-His antibody (1:1000) for one hour, and a final incubation with secondary Anti-mouse-Alexa488 (1:5000) for one hour, before dynabead release. Dynabeads transferred to a 96-well non-binding Greiner black plate were assessed for fluorescence of Alexa488 (495 nm excitation and emission at 519 nm) measured on a POLARstar OMEGA plate reader (BMG Labtech). Alternatively His-tag isolation and pulldown dynabeads were used to capture malE-en2NTS_1_ or en2NTS_1_ for one hour. Ligand binding was assessed through binding of the Alexa647 labelled NT_8–13_^50^ and competition assessed by introducing unlabelled NT_8–13_. Alexa647 fluorescence (650 nm excitation and emission at 668 nm) were also measured on a POLARstar OMEGA plate reader.

#### NMR Spectroscopy

Purified ^13^CH_3_-Methionine labelled malE-en2NTS_1_ or en2NTS_1_ was exchanged in NMR buffer (20 mM Na_2_HPO_4_-NaH_2_PO_4_ pH 7.5, 50 mM NaCl, 10% D_2_O) and placed into 5 mm NMR sample tubes. The NMR buffer included detergent when required (1 mM DDM, 1 mM LMPG). NMR spectra malE-en2NTS_1_ were acquired on approximately 20 µM malE-en2NTS_1_ ± 100 µM NT_8–13_, or 30 µM en2NTS_1_ ± 50 µM NT_8–13_ with a Bruker Avance 800 MHz spectrometer equipped with a cryogenic probe. 2D ^1^H-^13^C SOFAST-HMQC spectra^51^ were typically recorded at 298 K with spectral widths of 9,615 Hz (1,024 data points) and 8,000 Hz (128 data points) for the ^1^H and ^13^C dimensions respectively with a relaxation delay of 400 ms. The ^13^C carrier frequency was positioned at 17 ppm, and the ^1^H at 4.7 ppm, while band selective ^1^H pulses were centred at 2 ppm. Prior to Fourier transformation, data were multiplied by cosine-bells and zero-filled once in each dimension. All NMR data was processed in NMRPipe^52^ and plotted in Sparky (Goddard, T.D. and Kneller, D.G., University of California, San Francisco).

## Electronic supplementary material

Supplementary
