# Supplementary material for: Characterisation of a cell-free synthesised G-protein coupled receptor
Source: Sci Rep. 2017 Apr 24;7:1094. doi: 10.1038/s41598-017-01227-z (PMC5430785; doi:10.1038/s41598-017-01227-z)
Supplement: Supplementary file 1 — Supplementary [file 41598_2017_1227_MOESM1_ESM.pdf]

Supplementary Information

Characterisation of a cell-free synthesised G-protein coupled receptor

Patrick J. Shilling[1,2#*], Fabian Bumbak[1,2,3], Daniel Scott[1,3], Ross A.D. Bathgate[1,3], Paul R. Gooley[1,2*]

[1]Department of Biochemistry and Molecular Biology, The University of Melbourne, VIC 3010, Australia.

[2]Bio21 Molecular Science and Biotechnology Institute, 30 Flemington Road, The University of Melbourne, VIC 3010, Australia.

[3]The Florey Institute of Neuroscience and Mental Health, 30 Royal Parade, The University of Melbourne, Parkville, 3052 Victoria, Australia.

# Current address Department of Biochemistry and Biophysics, Stockholm University, Stockholm Sweden.

* To whom correspondence should be addressed: patrick.shilling@dbb.su.se and prg@unimelb.edu.au

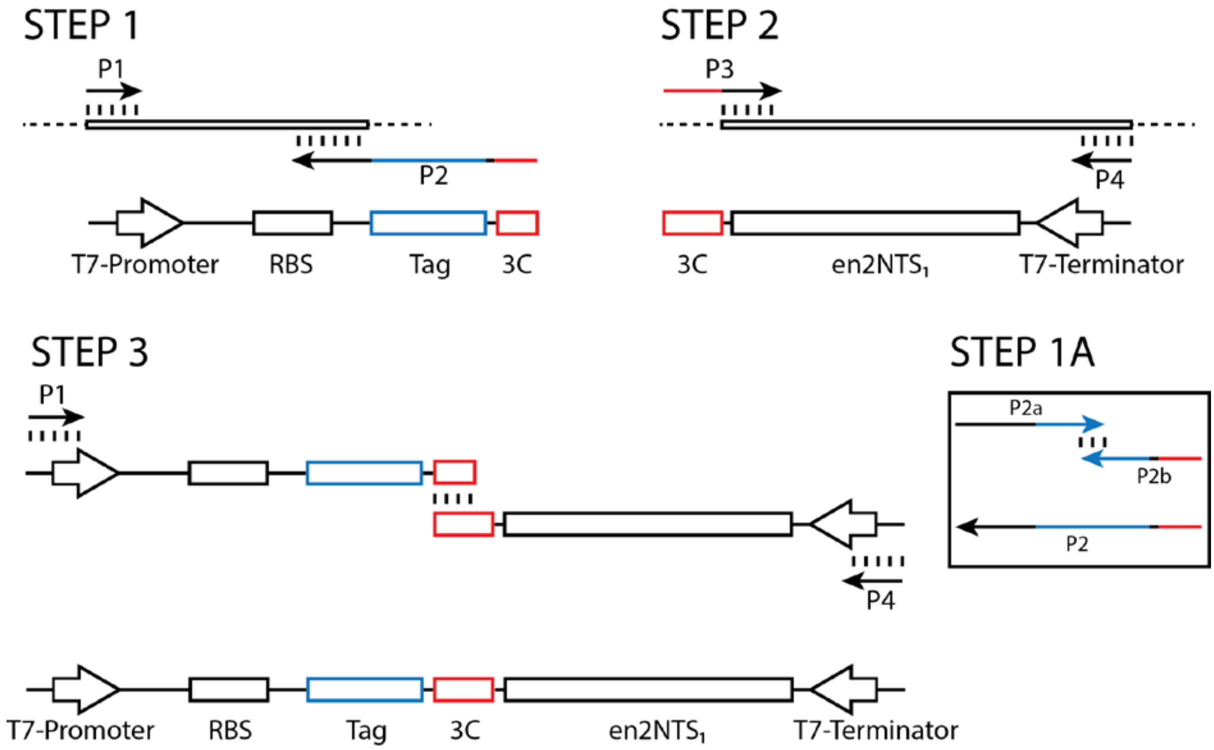

Figure S1. Overlap PCR strategy of en2NTS$_1$. Overlap PCR was used for quick generation of linear PCR fragments that could be used to determine which expression tag would be suitable for CFPS of en2NTS$_1$. Step 1 produced the T7P-RBS-Tag-3C fragment. Step 2 produced the 3C-en2NTS$_1$-T7T fragment. The products from step1 and 2 were used for a third overlap PCR which generated the full length CF expression template. Step 1A was undertaken prior to step 1 for the larger expression tags, ompA, ompC and malE.

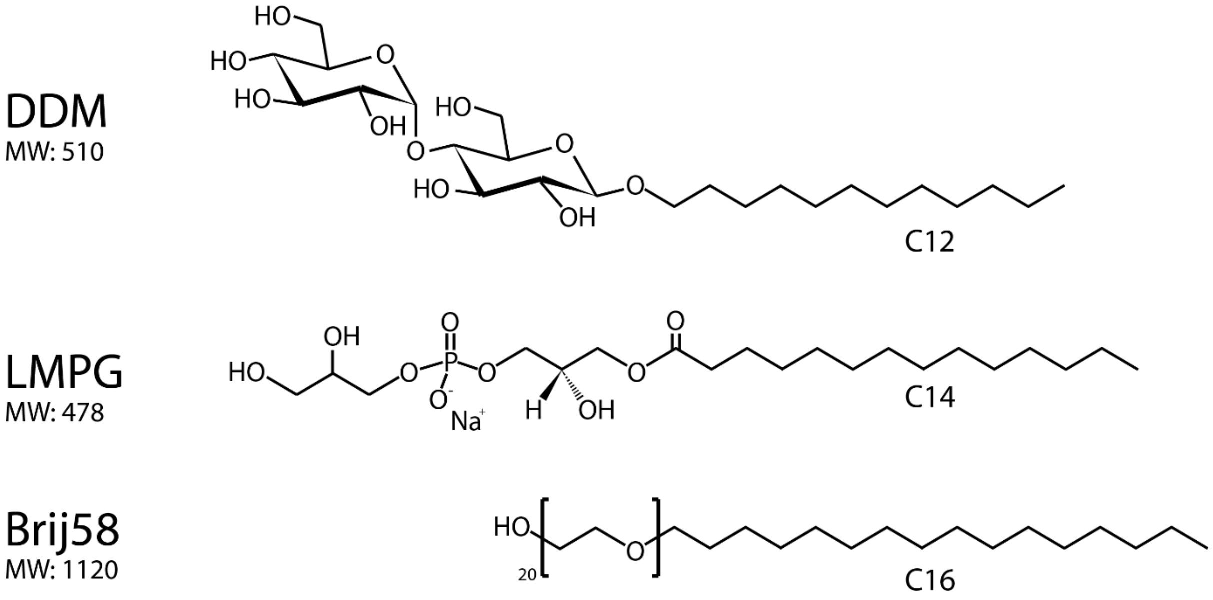

Figure S2. Monomeric compound structure of DDM, LMPG, and Brij58. DDM, LMPG, and Brij58 have differing alkyl chain lengths that affect the aggregation number and therefore average micelle size. The head groups also differ and can affect the interaction to a membrane protein.

```
                     10         20         30         40         50         60         70         80         90        100        110
rNTS1        MHLNSSVPQG TPGEPDAQPF SGPQSEMEAT FLALSLSNGS GNTSESDTAG PNSDLDVNTD IYSKVLVTAI YLALFVVGTV GNSVTAFTLA RKKSLQSLQS TVHYHLGSLA
en2NTS1      ---------- ---------- ---------- ---------- --TSESDTAG PNSDLDVNTD IYSKVLVTAI YLALFVVGTV GNGVTLFTLA RKKSLQSLQS RVDYYLGSLA
malE-en2NTS1 ------MKIK TGARILALSA LTTMMFSASA LAGSLEVLFQ GPTSESDTAG PNSDLDVNTD IYSKVLVTAI YLALFVVGTV GNGVTLFTLA RKKSLQSLQS RVDYYLGSLA
OGG7-ΔIC3    ---------- ---------- ---------- ---------- -----GPGSG PNSDLDVNTD IYSKVLVTAI YLALFVVGTV GNGVTLFTLA RKKSLQSLQS RVDYYLGSLA
                    120        130        140        150        160        170        180        190        200        210        220
rNTS1        LSDLLILLLA MPVELYNFIW VHHPWAFGDA GCRGYYFLRD ACTYATALNV ASLSVERYLA ICHPFKAKTL MSRSRTKKFI SAIWLASALL AIPMLFTMGL QNRSGDGTHP
en2NTS1      LSSLLILLFA LPVDVYNFIW VHHPWAFGDA GCKGYYFLRE ACTYATALNV VSLSVERYLA ICHPFKAKTL MSRSRTKKFI SAIWLASALL SLPMLFTVGL QNLSGDGTHP
malE-en2NTS1 LSSLLILLFA LPVDVYNFIW VHHPWAFGDA GCKGYYFLRE ACTYATALNV VSLSVERYLA ICHPFKAKTL MSRSRTKKFI SAIWLASALL SLPMLFTVGL QNLSGDGTHP
OGG7-ΔIC3    LSDLLILLFA LPVDVYNFIW VHHPWAFGDA GCKGYYFLRE ACTYATALNV VSLSVELYLA IRHPFKHKTL MSRSRTKKFI SAIWLASALL AIPMLFTVGL QNLSGDGTHP
                    230        240        250        260        270        280        290        300        310        320        330
rNTS1        GGLVCTPIVD TATVKVVIQV NTFMSFLFPM LVISILNTVI ANKLTVMVHQ AAEQGRVCTV GTHNGLEHST FNMTIEPGRV QALRHGVLVL RAVVIAFVVC WLPYHVRRLM
en2NTS1      GGLVCTPIVD TATLRVVIQL NTFMSFLFPM LVASILNTVI ARRLTVMVHQ AAEQARVSTV GTHNGLEHST FNMTIEPGRV QALRRGVLVL RAVVIAFVVC WLPYHVRRLM
malE-en2NTS1 GGLVCTPIVD TATLRVVIQL NTFMSFLFPM LVASILNTVI ARRLTVMVHQ AAEQARVSTV GTHNGLEHST FNMTIEPGRV QALRRGVLVL RAVVIAFVVC WLPYHVRRLM
OGG7-ΔIC3    GGLVCTPIVD TATLKVVIQL NTFMSFLFPM LVASILNTVI ARRLTVMVHQ AAEQGRVCT- ---------- -----EPGRV QALRRGVLVL RAMVIAFVVC WLPYHVRRLM
                    340        350        360        370        380        390        400        410        420
rNTS1        FCYISDEQWT TFLFDFYHYF YMLTNALFYV SSAINPILYN LVSANFRQVF LSTLACLCPG WRHRRKKRPT FSRKPNSMSS NHAFSTSATR ETLY--
en2NTS1      FVYISDEQWT TALFDFYHYF YMLSNALVYV SAAINPILYN LVSANFRQVF LSTLASLSPG WRHRRKKRPT FSRKPNSMSS NHAFST---- ------
malE-en2NTS1 FVYISDEQWT TALFDFYHYF YMLSNALVYV SAAINPILYN LVSANFRQVF LSTLASLSPG WRHRRKKRPT FSRKPNSMSS NHAFSTHHHH HHHHHH
OGG7-ΔIC3    FVYISDEQWT TALFDFYHYF YMLSNALVYV SAAINPILYN LVSANFRQVF LSTLACLCPG TRELEVLFQ- ---------- ---------- ------
```

Figure S3. Comparison of the protein sequence of wild type rat NTS_1 and two thermostabilised NTS_1 variants. The rat NTS_1 ($rNTS_1$) was the basis for the original thermostabilisation. Shown in red are the specific thermostabilising mutations that were created by the CHESS methodology, while in yellow are the $en2NTS_1$ specific modifications necessary for *E. coli* expression. $en2NTS_1$ was the progenitor for malE-$en2NTS_1$, however it lacks the components required for recombinant *E. coli* expression and instead possesses the N-terminal malE signal sequence and C-terminal His-tag in blue. The NTS_1 OGG7-ΔIC3 (4BV0) is a recently crystalised thermostabilised variant. It possesses many of the same thermostabilising mutations as $en2NTS_1$ and malE-$en2NTS_1$. The shortening of the N- and C-termini, deletion of intracellular loop 3, along with the variant mutations in relation to $en2NTS_1$ and malE-$en2NTS_1$ allow thermostability in the short chained detergent octyl-glucoside (OG).

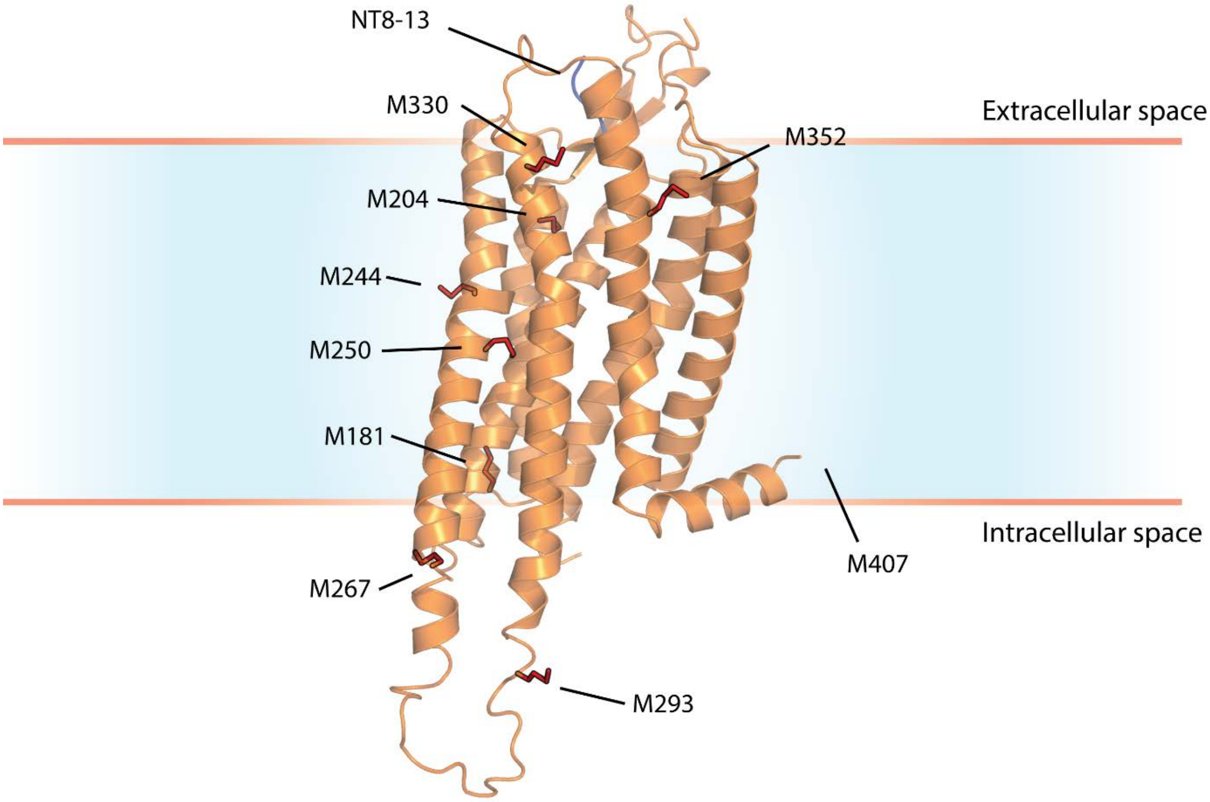

Figure S4. Methionine positions within malE-en2NTS$_1$. Nine methionines (red) are present in malE-en2NTS$_1$ and are highlighted for the pdb structure 4BV0. A further three methionines are found in the expression tag malE (not shown here). NT$_{8\text{-}13}$ is labelled and appears in blue. Several methionines are in close proximity to the ligand binding site and may be a good indicator for measuring interaction between NT$_{8\text{-}13}$ and en2NTS$_1$ by ^{1}H-^{13}C NMR.

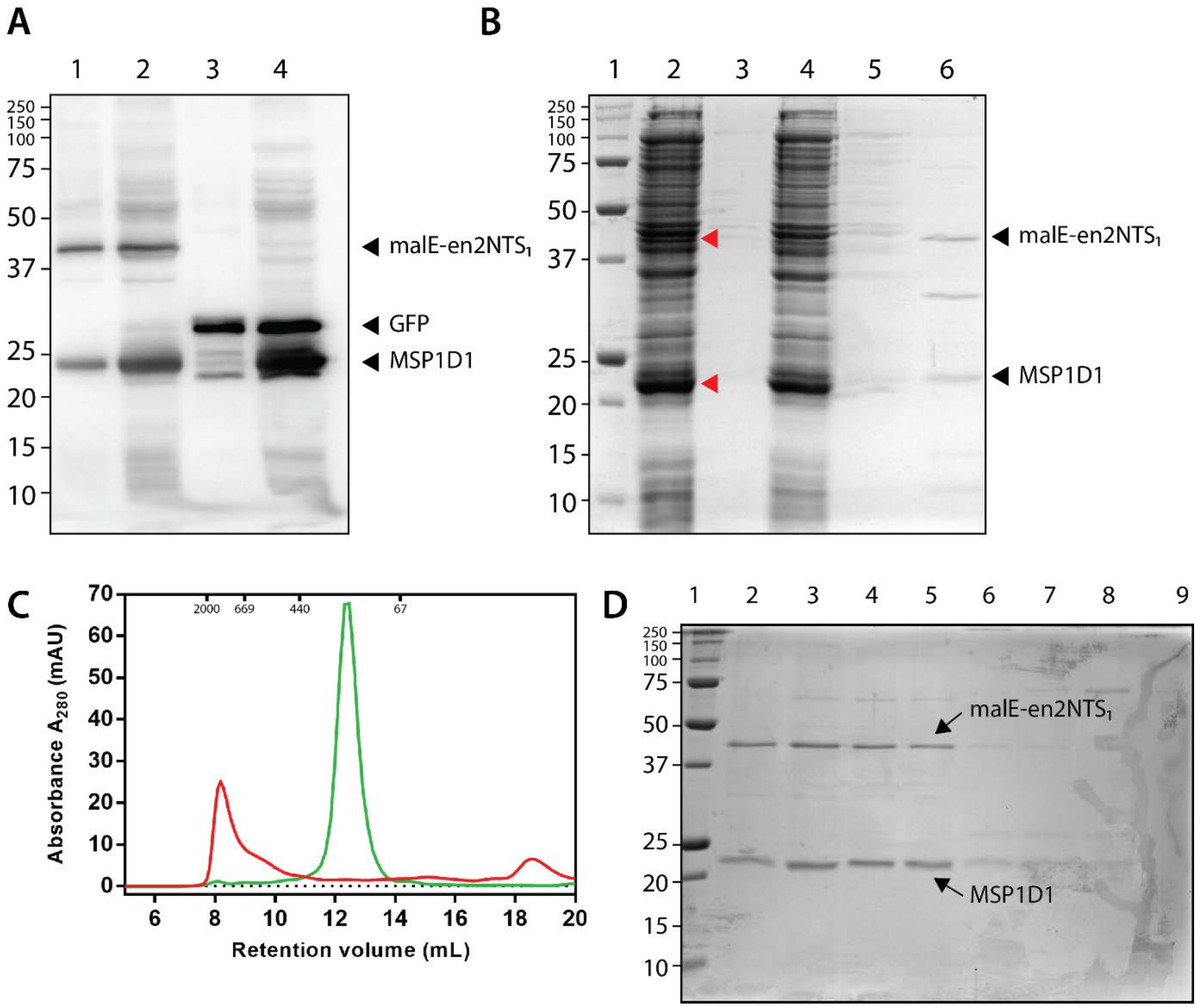

Figure S5. Expression tests and purification of ND-CF expressed malE-enNTS$_1$.
A. Immunoblot blot of expression tests with POPC ND. Anti-his-tag antibody was used for detection. **Lane 1 (L1):** malE-enNTS$_1$/ ND pellet, **L2:** malE-en2NTS$_1$/ ND supernatant, **L3:** GFP/ND pellet, **L4:** GFP/ ND supernatant. malE-en2NTS$_1$, GFP and MSP1D1 are marked. **B.** SDS-PAGE of expression and purification of malE-en2NTS$_1$-POPC nanodiscs. **L1:** Molecular weight marker, **L2:** Post CF supernatant; the bands of malE-en2NTS$_1$ and MSP1D1 are marked by red arrows; **L3:** Post CF pellet, **L4:** Ni-IMAC flow through, **L5:** Ni-IMAC wash, **L6:** Ni-IMAC elution.

The malE-en2NTS$_1$ nanodisc sample post NMR was purified by SEC **(C)** and eluted at the void volume (8 mL, Red line) suggestive of a very large complex. The catalase elution (green line) is used as a comparison of expected size of empty nanodiscs using the MSP1D1 scaffold protein. Calibration standards are marked at the top of the graph from highest molecular weight (kDa) to the smallest (blue dextran, thyroglobulin, ferritin and BSA). **(D)** SDS-PAGE of SEC fractions from **Lane 2 (L2)** 7.5 mL, **L3:** 8.0 mL, **L4:** 8.5 mL, **L5:** 9.0 mL, **L6:** 9.5 mL, **L7:** 10.0 mL, **L8:** 10.5 mL, **L9:** 11.0 mL. The molecular weight marker, malE-en2NTS$_1$ and MSP1D1 are marked.

Table S1. Primers for linear PCR expression templates.

Primer abbreviation	Overlap PCR primer type	Primer name	5’ - 3’
P1	P1	T7[a]-Forward flank	GAGATCTCGATCCCGCGAAA
P2	P2	RBS[b]-MASMTG-3C[c]	CTGGAACAGAACTTCCAGGGATCCACCGGTCATAGAAGCCATATGTATATCTCC
P3	P2	RBS-AT-3C	CTGGAACAGAACTTCCAGGGATCCATAATATTTATAATATTTCATATGTATATCTCC
P4	P2	RBS-SER-3C	CTGGAACAGAACTTCCAGGGATCCTGATGATGATGATGATTTCATATGTATATCTCC
P5	P2	RBS-H-3C	CTGGAACAGAACTTCCAGGGATCCTGGACCATCGTATGGTTTCATATGTATATCTCC
P6	P2	RBS-G-3C	CTGGAACAGAACTTCCAGGGATCCTTCTTCTCCTTTACTTTTCATATGTATATCTCC
P7	P2	RBS-HA-3C	GAACTTCCAGGGATCCAGCGTAATCTGGAACATCGTATGGGTACATATGTATATCTCC
P8	P2a	malE FWD*	GGAGATATACATATGAAAATAAAAACAGGTGCACGCATCCTCGCATTATCCGCATTAACGA
P9	P2b	malE RVS*	GAACTTCCAGGGATCCGGCGAGAGCCGAGGCGGAAAACATCATCGTCGTTAATGCGGA
P10	P2a	ompA FWD*	GGAGATATACATATGAAAAAGACAGCTATCGCGATTGCAGTGGCACTGGCTG
P11	P2b	ompA RVS*	GAACTTCCAGGGATCCGGCCTGCGCTACGGTAGCGAAACCAGCCAGTGCCA
P12	P2a	ompC FWD*	GGAGATATACATATGAAAGTTAAAGTACTGTCCCTCCTGGTCCCAGCTCTGCT
P13	P2b	ompC RVS*	GAACTTCCAGGGATCCAGCGTTTGCTGCGCCTGCTACCAGCAGAGCTGGG
P14	P3	3C-en2NTS$_1$-FWD	GGATCCCTGGAAGTTCTGTTCCAGGGGCCCACCTCTGAATCTGACACC
P15	P4	T7-Reverse flank	GGATATAGTTCCTCCTTTCAGC

* Indicates an overlap PCR (STEP 1A) was required to generate the expression tag prior to STEP 1 – Figure S1
[a] T7 – T7 promoter and terminator sites of T7RNA polymerase
[b] RBS – Ribosome binding site
[c] 3C – 3C protease site
